# Longitudinal study on ultrasound assessment of peripheral arterial elasticity and liver stiffness for evaluating clinical intervention efficacy in children with obesity

**DOI:** 10.3389/fped.2025.1604576

**Published:** 2025-11-21

**Authors:** Ning Zheng, Qiaosheng Jiang, Lizhen Zou, Xiaodan Fang, Yunjiao Zhang

**Affiliations:** 1 Department of Ultrasound, Jinhua people’s Hospital of Zhejiang, Jinhua, Zhejiang, China; 2 Department of Radiology, Jinhua Central Hospital, Jinhua, Zhejiang, China; 3 Department of Pediatrics, Jinhua people’s Hospital of Zhejiang, Jinhua Zhejiang, China

**Keywords:** children with obesity, vascular elasticity, liver stiffness, ultrasound, clinical intervention

## Abstract

**Objective:**

To evaluate the efficacy of a multidisciplinary clinical intervention in children with obesity by longitudinally monitoring changes in vascular elasticity and liver stiffness via ultrasound-derived parameters.

**Methods:**

A single-center longitudinal study was conducted on 120 children with obesity (aged 7–14 years, BMI ≥95th percentile). The participants underwent a 12-month intervention involving personalized low-calorie diets, structured aerobic exercise, and family-based cognitive–behavioral therapy. Vascular parameters (carotid intima–media thickness [IMT], flow-mediated dilation [FMD], elasticity coefficient [Ep], stiffness index [β], distensibility coefficient [CD], arterial compliance [AC]) and liver stiffness [shear wave velocity (SWV)] were assessed at baseline (T0), 6 months (T1), and 12 months (T2) via Siemens S2000 ultrasound with ARFI technology. Statistical analyses included repeated-measures ANOVA, Pearson correlation, and multivariate linear regression.

**Results:**

Significant improvements were observed in FMD (7.1%–9.5%, *P* < 0.001), Ep (60.5 kPa–42.3 kPa, *P* < 0.001), β (6.25–4.55, *P* < 0.001), CD (10.05 kPa–14.9 kPa, *P* < 0.001), AC (0.96–1.29 mm^2^/kPa, *P* < 0.001), and SWV (1.36 m/s–0.98 m/s, *P* < 0.001). The carotid IMT did not significantly change (*Δ* = −0.01 mm, *P* = 0.16). Multivariate regression identified *Δ*BMI as an independent predictor of *Δ*FMD (*β* = 0.32, *P* = 0.003) and *Δ*SWV (*β* = −0.41, *P* < 0.001).

**Conclusion:**

Ultrasound-based multiparameter assessment effectively quantifies improvements in vascular and hepatic function following clinical intervention in children with obesity.

## 1 Introduction

The global epidemic of childhood obesity represents a major public health challenge, and its long-term associations with cardiovascular diseases and metabolic dysfunction-associated steatotic liver disease (MASLD) have drawn significant attention (1). According to the China Childhood Obesity Report, the prevalence of overweight and obesity among school-aged children in China is projected to reach 28% by 2030 without timely intervention (2). Quantitative evidence demonstrates that obesity induces early tissue remodeling in multiple organ systems. For example, shear wave elastography has revealed 23% higher meniscal stiffness in children with obesity than in their healthy-weight peers, indicating accelerated connective tissue degeneration (3). Children with obesity face not only early risks of atherosclerosis and hepatic steatosis but also significantly elevated rates of cardiovascular events and liver fibrosis in adulthood (4). Therefore, exploring effective tools for monitoring intervention outcomes is critical for delaying or reversing obesity-related complications.

Currently, the gold standard for assessing childhood obesity complications relies heavily on invasive procedures such as liver biopsy and angiography, yet their traumatic nature and operational risks limit their clinical application (5). Although carotid intima‒media thickness (IMT) and flow-mediated dilation (FMD) are widely used to evaluate vascular health (6), single parameters struggle to comprehensively reflect the complexity of arterial elasticity changes. Similarly, while liver stiffness measurement via transient elastography (FibroScan) is feasible (7), its accessibility and cost-effectiveness in pediatric populations remain controversial. In recent years, acoustic radiation force impulse (ARFI) imaging has emerged as a novel noninvasive and quantitative option for pediatric liver stiffness assessment (8), and multiparametric ultrasound analysis (e.g., elasticity coefficient Ep and stiffness β) offers new insights into dynamic vascular function monitoring (9). However, existing studies have focused predominantly on single-organ evaluations and lack systematic exploration of synergistic changes along the vascular‒liver axis.

This study employs a prospective cohort design to investigate dynamic changes in vascular and hepatic function among children with obesity undergoing multidisciplinary interventions, utilizing multiparametric ultrasound to establish quantitative associations between weight control and multisystem improvement.

## 2 Materials and methods

### 2.1 Study design

This non-randomized, self-controlled longitudinal study was designed to track within-participant changes following a standardized clinical intervention. Randomization was not employed as all eligible participants received the multidisciplinary intervention per hospital protocol. This single-center longitudinal study was conducted at the Pediatric Outpatient Department of Zhejiang Jinhua Municipal People's Hospital from March 2022 to December 2023. The study protocol was approved by the Ethics Committee of Zhejiang Jinhua Municipal People's Hospital (Approval No. 2023068). Given that the study involved only routine clinical interventions and noninvasive ultrasound monitoring, the ethics committee waived informed consent requirements. However, all participants and their guardians signed anonymized data usage authorization forms. The study adhered to the principles of the Declaration of Helsinki, with clinical data collected via a standardized electronic medical record system and patient privacy rigorously protected.

This single-center longitudinal study was conducted at … patient privacy rigorously protected. Given the ethical constraints of withholding clinical intervention from children with obesity and the longitudinal nature of vascular maturation in children, a non-intervention control group was not included. To address potential confounding by natural growth, we compared observed changes with established pediatric reference values for vascular parameters and utilized repeated measurements to mitigate regression-to-mean effects.

To address missing data, multiple imputation (5 imputed datasets) was applied for continuous variables under the missing-at-random assumption, with baseline characteristics and adherence metrics as predictors. All analyses followed intention-to-treat principles.

### 2.2 Inclusion and exclusion criteria

Inclusion criteria:

1. Age 7–14 years, meeting the Working Group on Obesity in China criteria (BMI ≥95th percentile) (10);
2. Metabolic dysfunction-associated steatotic liver disease (MASLD) was confirmed by ultrasound according to the imaging criteria outlined in the Chinese Society of Hepatology Guidelines for Nonalcoholic Fatty Liver Disease (11).

Exclusion criteria:

1. Secondary obesity (e.g., endocrine disorders, genetic syndromes);
2. Comorbid diabetes, chronic liver diseases (viral hepatitis, autoimmune liver disease), or cardiovascular diseases (hypertension, congenital heart defects);
3. Use of metabolism- or weight-affecting medications (e.g., glucocorticoids, antipsychotics) within the past 6 months;
4. Severe systemic diseases or inability to comply with follow-up.

### 2.3 Interventions

All enrolled children with obesity received a 12-month multidisciplinary intervention.

Dietary intervention: Registered dietitians developed personalized low-calorie diet plans on the basis of individual basal metabolic rates and dietary habits, reducing total daily caloric intake by 20% while optimizing macronutrient composition (increased dietary fiber, high-quality protein, and unsaturated fats; restricted added sugars and saturated fats). Compliance was monitored weekly via food diaries and nutritional analysis software, with dietary adherence quantified as: (1) proportion of days with completed food diaries (target ≥5 days/week), and (2) percentage of weeks meeting caloric targets (±10% of prescribed intake).

Exercise intervention: Rehabilitation specialists designed moderate-intensity aerobic exercise programs (e.g., brisk walking, swimming, cycling) that were conducted 5 times weekly (60 min/session), targeting heart rates ≥140 bpm. Exercise intensity was progressively adjusted according to baseline fitness levels, with supervision by professional coaches to ensure safety and adherence. Exercise adherence was quantified as session attendance rate (sessions attended/total prescribed sessions), with target adherence set at ≥80%.

Behavioral therapy: Family-involved cognitive behavioral interventions included monthly group sessions covering self-monitoring skills (e.g., diet/exercise logging), goal setting, positive reinforcement strategies, and stress management. Interactive methods (role-playing, simulations) were supplemented with homework (e.g., “Healthy Challenge Week”) to reinforce behavioral changes. Parents participated in all the sessions and adjusted their home environments (e.g., reducing high-calorie snack availability). Additionally, the research team provided personalized guidance via monthly phone calls and instant messaging (WeChat) to address adherence issues (Supplementary Table S1).

### 2.4 Data collection and measurements

Data collection was conducted at three time points: baseline (T0, preintervention), 6 months postintervention (T1), and 12 months postintervention (T2). All measurements were performed by uniformly trained medical staff following standardized protocols to minimize interoperator variability.

**Figure 1:**
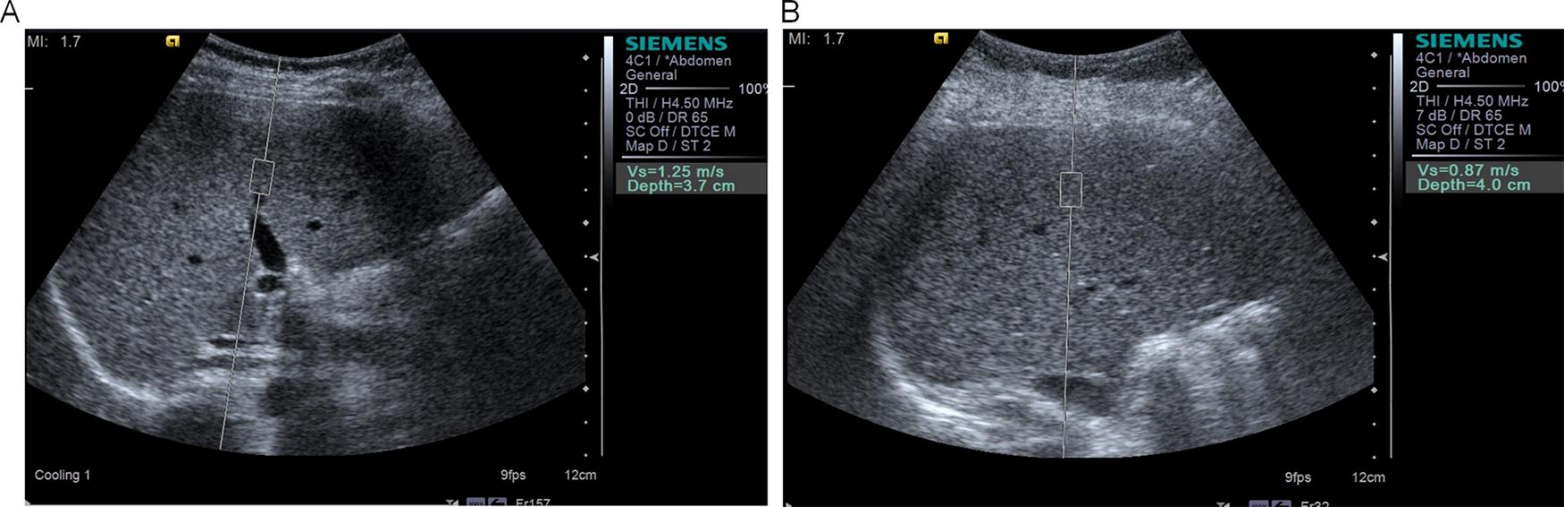
Acoustic radiation force impulse (ARFI) elastography of liver in study participants. **(A)** Representative image showing liver stiffness measurement in an obese child with SWV of 1.25 m/s, indicating increased liver stiffness. **(B)** Representative image showing liver stiffness measurement in a normal-weight child with SWV of 0.87 m/s, demonstrating normal liver elasticity. SWV, shear wave velocity.

Ultrasound parameters: All ultrasound examinations were performed by a senior radiologist with over 10 years of experience in pediatric vascular ultrasonography. The examiner was certified by the Chinese Medical Doctor Association and the Chinese Society of Ultrasound Medicine, trained per standardized protocols (12). Measurements followed technical guidelines: (1) Fasting >3 h; (2) Supine position with right arm maximally abducted; (3) Probe perpendicular to liver capsule with light pressure; (4) Sampling depth 3–6 cm below Glisson's capsule, avoiding vessels >3 mm. A Siemens S2000 color Doppler ultrasound system (linear probe frequency: 10 MHz; convex probe frequency: 4.5 MHz) was used to measure the following parameters: carotid IMT: participants were placed in the supine position, and the IMT was measured at 12 sites (bilateral common carotid artery, bifurcation, and proximal/distal internal carotid artery), with the mean value calculated. FMD: The brachial artery was selected as the target vessel. The baseline diameter (Bd) and postreactive hyperemia diameter (Fd) were measured, and FMD was calculated as (Fd−Bd)/Bd × 100%. Arterial elasticity parameters: Systolic pressure (Ps), diastolic pressure (Pd), and carotid artery diameter changes (Ds, Dd) were synchronously recorded to calculate the elasticity coefficient (Ep), stiffness β, distensibility coefficient (DC), and arterial compliance (AC). Liver stiffness measurement: Shear wave velocity (SWV) was measured using acoustic radiation force impulse (ARFI) imaging. Five measurements were taken in the right anterior liver lobe (while avoiding vascular structures), and the mean value was calculated and reported in m/s. Young's modulus (E) was calculated from SWV using the formula represents SWV (12), with results expressed in kilopascals (kPa). The representative image of liver elasticity measured by ARFI technology is shown in Figure 1. Carotid artery ultrasound measurements for vascular elasticity assessment were performed as shown in Supplementary Figure S1. The methodology for obtaining carotid artery parameters, including intima-media thickness and blood flow velocity measurements, is illustrated in Supplementary Figure S2.

To assess measurement reliability, intra-observer and inter-observer reproducibility were evaluated for critical ultrasound parameters in a subset of 30 randomly selected participants who underwent duplicate scans by the same operator and a second blinded operator within 48 h.

Clinical parameters: Weight and height: measured via a calibrated electronic stadiometer (model: SECA 284), with BMI calculated as weight (kg)/height (m^2^); blood pressure: measured three times via a standard cuff sphygmomanometer (Omron HEM-7121), with the mean value recorded; and lipid profile: fasting venous blood samples were analyzed for total cholesterol (TC), low-density lipoprotein cholesterol (LDL-C), and high-density lipoprotein cholesterol (HDL-C) via the hospital's central laboratory.

Data management and quality control: All the data were entered in real time via an electronic medical record (EMR) system, with dual independent verification to ensure accuracy. Ultrasound images and raw data were archived for review. Lipid measurements were validated through internal laboratory quality controls (coefficient of variation <5%).

Blinding procedures: Ultrasound operators were blinded to (1) participant identities (assigned anonymized IDs), (2) intervention time points (scans labeled only with study codes), and (3) clinical parameters (blood pressure/lipid results). Image analyses were performed on de-identified datasets using standardized protocols.

### 2.5 Statistical analysis

Data analysis was performed via SPSS 26.0. Missing data were handled via multiple imputation using chained equations (MICE) with 5 iterations, incorporating baseline covariates and observed follow-up data to preserve statistical power and reduce bias. Continuous variables are expressed as the mean ± standard deviation (SD). The primary analysis employed paired t tests to compare changes in ultrasound parameters and clinical indicators pre- and postintervention, with a significance threshold of *P* < 0.05. The secondary analysis utilized multiple linear regression models, where *Δ*FMD and *Δ*SWV served as dependent variables and where *Δ*BMI and *Δ*systolic/diastolic blood pressure were independent variables. Adherence-outcome associations were assessed via linear regression models, with dietary/exercise adherence rates as covariates in sensitivity analyses of *Δ*FMD and *Δ*SWV. Adjustments were made for age, sex, and other potential confounders to evaluate independent associations (β coefficients and *P* values). Multicollinearity among independent variables (*Δ*BMI, *Δ*SBP, *Δ*DBP, *Δ*LDL-C) was assessed using variance inflation factors (VIF). The sensitivity analysis excluded 15 participants with postintervention weight rebound (BMI increase ≥5%) to verify the robustness of the results. All the statistical tests were two-tailed. Data normality was assessed via the Shapiro‒Wilk test, with nonnormal data analyzed via nonparametric tests (Mann‒Whitney *U*).

## 3 Results

### 3.1 Baseline characteristics

During the 12-month intervention period, 12 participants (10.0%) were lost to follow-up due to relocation (*n* = 7), withdrawal of consent (*n* = 3), or inability to comply with scheduled visits (*n* = 2). The study enrolled 120 children with obesity, including 68 males (56.7%) and 52 females (43.3%). Baseline clinical and ultrasound parameters revealed a mean BMI of 28.4 ± 3.1 kg/m^2^ and systolic and diastolic blood pressures of 125 ± 8 mmHg and 78 ± 6 mmHg, respectively, with total cholesterol (TC) and LDL-C levels exceeding normal pediatric reference ranges. Ultrasound measurements revealed an IMT of 0.58 ± 0.05 mm, FMD of 7.1 ± 2.3%, and SWV of 1.36 ± 0.23 m/s. Gender comparisons revealed no statistically significant differences in BMI, blood pressure, or ultrasound parameters between males and females (*P* > 0.05), confirming baseline homogeneity (Table 1).

**Table 1:** Baseline characteristics of the study cohort.

Parameter	Total cohort (*n* = 120)	Male (*n* = 68)	Female (*n* = 52)	Statistic	*P* value
Demographics					
Age (years)	9.2 ± 1.5	9.4 ± 1.6	9.0 ± 1.4	*t* = 1.34	0.18
Clinical Parameters					
BMI (kg/m^2^)	28.4 ± 3.1	28.6 ± 3.3	28.1 ± 2.8	*t* = 0.93	0.35
Systolic BP (mmHg)	125 ± 8	126 ± 9	124 ± 7	*t* = 1.23	0.22
Diastolic BP (mmHg)	78 ± 6	79 ± 7	77 ± 5	*t* = 1.61	0.11
TC (mmol/L)	4.8 ± 0.9	4.7 ± 0.8	4.9 ± 1.0	*t* = −1.15	0.25
LDL-C (mmol/L)	3.1 ± 0.7	3.0 ± 0.6	3.2 ± 0.8	*t* = −1.70	0.09
Ultrasonic Parameters					
Carotid IMT (mm)	0.58 ± 0.05	0.57 ± 0.06	0.59 ± 0.04	*t* = −1.83	0.07
FMD (%)	7.1 ± 2.3	7.3 ± 2.5	6.8 ± 2.1	*t* = 1.08	0.28
Ep (kPa)	60.5 ± 11.1	61.2 ± 10.8	59.7 ± 11.5	*t* = 0.76	0.45
β	6.25 ± 1.35	6.30 ± 1.40	6.18 ± 1.28	*t* = 0.48	0.63
CD (kPa)	10.05 ± 0.33	10.10 ± 0.30	9.98 ± 0.36	*t* = 1.91	0.06
AC (mm^2^/kPa)	0.96 ± 0.43	0.93 ± 0.41	1.00 ± 0.45	*t* = −0.88	0.38
SWV (m/s)	1.36 ± 0.23	1.38 ± 0.25	1.33 ± 0.20	*t* = 1.11	0.27

Data are presented as the means ± standard deviations. Statistical analysis: Independent *t* tests were used for all the parameters (Shapiro‒Wilk tests confirmed normality).

BMI, body mass index; BP, blood pressure; TC, total cholesterol; LDL-C, low-density lipoprotein cholesterol; IMT, intima–media thickness; FMD, flow-mediated dilation; Ep, elasticity coefficient; CD, distensibility coefficient; AC, arterial compliance; SWV, shear wave velocity.

### 3.2 Changes in ultrasound parameters postintervention

#### 3.2.1 Vascular structure and functional indicators

**Figure 2:**
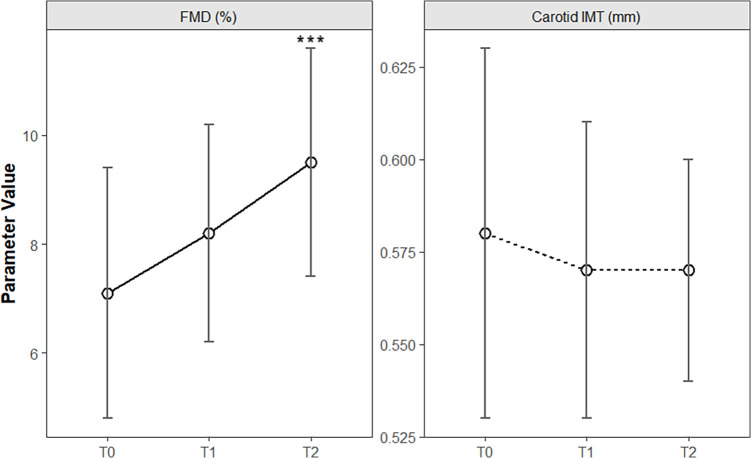
Temporal changes in vascular structural and functional parameters during clinical intervention in children with obesity. The line chart shows the longitudinal trends of carotid intima–media thickness (IMT) and flow-mediated dilation (FMD) at baseline (T0), 6 months (T1), and 12 months (T2) of intervention. The error bars represent the means ± standard deviations. ***P < 0.001.

Longitudinal analysis across baseline (T0), 6 months (T1), and 12 months (T2) demonstrated phased improvements in vascular structure and function. The IMT slightly decreased from 0.58 ± 0.05 mm at T0 to 0.57 ± 0.03 mm at T2, but the overall change lacked statistical significance [F(2, 118) = 1.85, *P* = 0.16]. In contrast, FMD significantly improved from 7.1 ± 2.3% at T0 to 8.2 ± 2.0% at T1 and 9.5 ± 2.1% at T2, with a significant time main effect [F(2, 118) = 22.34, *P* < 0.001]. *Post hoc* tests (Bonferroni-adjusted) confirmed significant differences between T0 and T1 (*P* = 0.003) and between T0 and T2 (*P* < 0.001), indicating early vascular endothelial function improvement by 6 months (Table 2 and Figure 2).

**Table 2:** Longitudinal changes in vascular structural and functional parameters.

Parameter	T0	T1	T2	Statistic	*P* value	Post hoc comparisons
IMT (mm)	0.58 ± 0.05	0.57 ± 0.04	0.57 ± 0.03	F(2, 118) = 1.85	0.16	T0 vs. T1: *P* = 0.23
FMD (%)	7.1 ± 2.3	8.2 ± 2.0	9.5 ± 2.1	F(2, 118) = 22.34	<0.001	T0 vs. T1: *P* = 0.003; T0 vs. T2: *P* < 0.001; T1 vs. T2: *P* = 0.04

Data are presented as the means ± standard deviations. Statistics: Repeated-measures ANOVA with Greenhouse–Geisser correction (sphericity violated for FMD, *ε* = 0.89). Post hoc: Bonferroni-adjusted pairwise comparisons.

IMT, Intima-media thickness; FMD, Flow-mediated dilation.

#### 3.2.2 Arterial elasticity indicators

**Figure 3:**
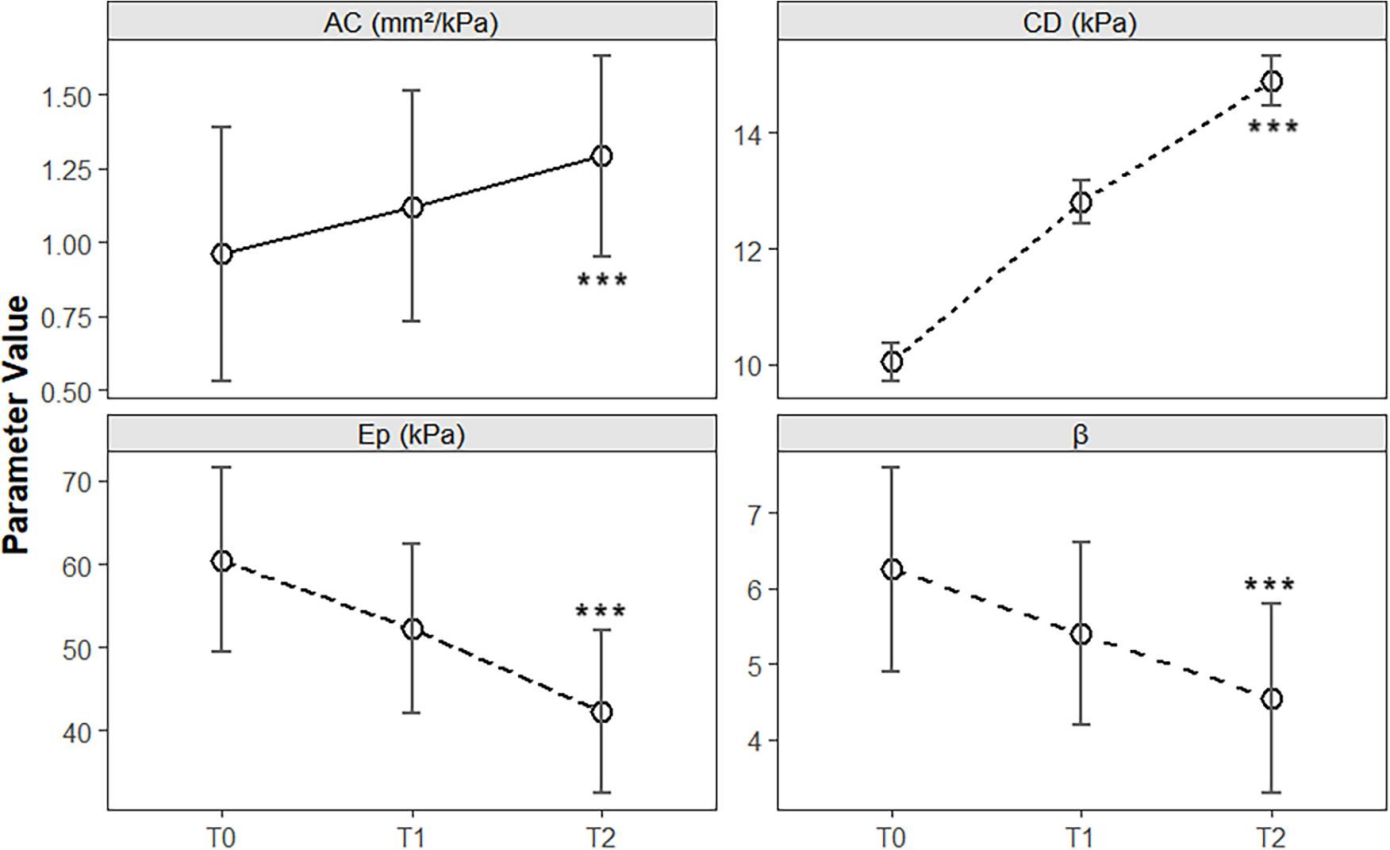
Longitudinal changes in arterial elasticity parameters during clinical intervention in children with obesity. The line chart illustrates the temporal trends of the elasticity coefficient (Ep), stiffness index (β), distensibility coefficient (CD), and arterial compliance (AC) at baseline (T0), 6 months (T1), and 12 months (T2) of multidisciplinary intervention. The error bars represent the means ± standard deviations. Significant improvements were observed in Ep, β, CD, and AC over the 12-month intervention period (see Table 3 for statistical details). ***P < 0.001.

Postintervention arterial elasticity parameters were markedly improved. Ep decreased from 60.5 ± 11.1 kPa at baseline to 52.3 ± 10.2 kPa at T1 and 42.3 ± 9.8 kPa at T2 [F(2, 118) = 38.72, *P* < 0.001]. β decreased from 6.25 ± 1.35 to 4.55 ± 1.25 at T2 [F(2, 118) = 24.15, *P* < 0.001], whereas CD and AC increased significantly from 10.05 ± 0.33 kPa to 14.9 ± 0.43 kPa [F(2, 118) = 112.6, *P* < 0.001] and 0.96 ± 0.43 mm^2^/kPa to 1.29 ± 0.34 mm^2^/kPa [F(2, 118) = 9.84, *P* < 0.001], respectively. *Post hoc* tests revealed significant improvements in Ep, β, and CD by T1 (*P* < 0.05), whereas AC improved only by T2 (Table 3 and Figure 3).

**Table 3:** Longitudinal changes in arterial elasticity parameters.

Parameter	T0	T1	T2	Statistic	*P* value	Post hoc comparisons
Ep (kPa)	60.5 ± 11.1	52.3 ± 10.2	42.3 ± 9.8	F(2, 118) = 38.72	<0.001	T0 vs. T1: *P* = 0.002; T0 vs. T2: *P* < 0.001; T1 vs. T2: *P* = 0.01
β	6.25 ± 1.35	5.40 ± 1.20	4.55 ± 1.25	F(2, 118) = 24.15	<0.001	T0 vs. T1: *P* = 0.03; T0 vs. T2: *P* < 0.001; T1 vs. T2: *P* = 0.04
CD (kPa)	10.05 ± 0.33	12.8 ± 0.38	14.9 ± 0.43	F(2, 118) = 112.6	<0.001	T0 vs. T1: *P* < 0.001; T0 vs. T2: *P* < 0.001; T1 vs. T2: *P* = 0.007
AC (mm^2^/kPa)	0.96 ± 0.43	1.12 ± 0.39	1.29 ± 0.34	F(2, 118) = 9.84	<0.001	T0 vs. T1: *P* = 0.12; T0 vs. T2: *P* < 0.001; T1 vs. T2: *P* = 0.04

Data are presented as the means ± standard deviations. Statistics: Repeated-measures ANOVA with Greenhouse–Geisser correction. Post hoc: Bonferroni-adjusted pairwise comparisons.

Ep, elasticity coefficient; β, stiffness index; CD, distensibility coefficient; AC, arterial compliance.

### 3.3 Liver stiffness changes

Liver SWV and derived Young's modulus (E) significantly decreased after intervention. The baseline SWV (1.36 ± 0.23 m/s) and E (5.54 ± 1.84 kPa) indicated elevated liver stiffness. After 6 and 12 months of intervention, SWV decreased to 1.12 ± 0.18 m/s (E: 3.76 ± 1.21 kPa) and 0.98 ± 0.12 m/s (E: 2.88 ± 0.71 kPa), respectively, with significant time main effects [SWV: F_(2118)_ = 48.15, *P* < 0.001; E: F_(2118)_ = 46.32, *P* < 0.001], confirming time-dependent improvements in liver stiffness (Table 4).

**Table 4:** Longitudinal changes in liver stiffness (SWV).

Parameter	T0	T1	T2	Statistic	*P* value	Post hoc comparisons
SWV (m/s)	1.36 ± 0.23	1.12 ± 0.18	0.98 ± 0.12	F(2, 118) = 48.15	<0.001	T0 vs. T1: *P* = 0.008; T0 vs. T2: *P* < 0.001; T1 vs. T2: *P* = 0.02
Young's modulus (kPa)	5.54 ± 1.84	3.76 ± 1.21	2.88 ± 0.71	F(2, 118) = 46.32	<0.001	T0 vs. T1: *P* = 0.01; T0 vs. T2: *P* < 0.001; T1 vs. T2: *P* = 0.03

Data are presented as the means ± standard deviations. Statistics: Repeated-measures ANOVA with Greenhouse–Geisser correction (*ε* = 0.91). Post hoc: Bonferroni-adjusted pairwise comparisons.

SWV, shear wave velocity.

### 3.4 Interparameter correlation analysis

Correlations between changes in vascular function (*Δ*FMD, *Δ*Ep, *Δ*β, *Δ*CD, and *Δ*AC) and liver stiffness (*Δ*SWV) were analyzed. The *Δ*FMD was significantly negatively correlated with the *Δ*SWV (*r* = −0.46, *P* < 0.001), suggesting that vascular endothelial improvement is associated with a reduction in liver stiffness. Additionally, *Δ*Ep (*r* = 0.38, *P* < 0.001) and *Δ*β (*r* = 0.32, *P* = 0.001) were positively correlated with *Δ*SWV. To account for potential confounding by weight change, partial correlation analyses adjusting for *Δ*BMI were performed. After adjustment, *Δ*FMD remained negatively correlated with *Δ*SWV (r_partial = −0.35, *P* = 0.001), while *Δ*Ep (r_partial = 0.28, *P* = 0.007) and *Δ*β (r_partial = 0.24, *P* = 0.02) retained positive correlations with *Δ*SWV. The associations for *Δ*CD (r_partial = −0.15, *P* = 0.15) and *Δ*AC (r_partial = −0.14, *P* = 0.18) became nonsignificant. These findings suggest that the deterioration of arterial elasticity parallels the progression of liver fibrosis (Table 5).

**Table 5:** Correlation analysis between vascular and hepatic parameters.

Variable pair	Correlation coefficient (*r*)	*P* value	Adjusted r_partial (*P*)
*Δ*FMD vs. *Δ*SWV	−0.46	<0.001	−0.35 (0.001)
*Δ*Ep vs. *Δ*SWV	0.38	<0.001	0.28 (0.007)
*Δ*β vs. *Δ*SWV	0.32	0.001	0.24 (0.02)
*Δ*CD vs. *Δ*SWV	−0.28	0.004	−0.15 (0.15)
*Δ*AC vs. *Δ*SWV	−0.22	0.020	−0.14 (0.18)

Pearson correlation analysis was performed for bivariate relationships after confirming normality of all *Δ*-parameters using Shapiro–Wilk tests (all *P* > 0.05). Partial correlations (r_partial) were calculated through linear regression models adjusting for *Δ*BMI to control for weight change effects.

*Δ*, change from baseline; FMD, flow-mediated dilation; Ep, elasticity coefficient; β, stiffness index; CD, distensibility coefficient; AC, arterial compliance; SWV, shear wave velocity.

### 3.5 Multivariate linear regression analysis

Multivariate models adjusted for multicollinearity (VIF <5 for all predictors) were used to assess the independent effects of weight change (*Δ*BMI) and clinical indicators on vascular and hepatic improvements (Table 6). *Δ*BMI independently predicted *Δ*FMD (*β* = 0.32, *P* = 0.003) and *Δ*SWV (*β* = −0.41, *P* < 0.001), indicating that weight loss directly enhances vascular repair and liver softening. *Δ*SBP independently influenced *Δ*Ep (*β* = 0.18, *P* = 0.04) and *Δ*β (*β* = 0.15, *P* = 0.03), suggesting that blood pressure control partially mitigated arterial stiffening (Table 7).

**Table 6:** Variance inflation factors (VIF) for independent variables in multivariate regression models.

Predictor	*Δ*FMD model	*Δ*SWV model	*Δ*Ep model	*Δ*β model
*Δ*BMI	1.82	1.95	1.78	1.80
*Δ*SBP	2.10	–	2.05	2.08
*Δ*DBP	1.92	–	1.88	1.90
*Δ*LDL-C	1.75	–	1.72	1.74

VIF was computed for each predictor in the respective linear regression models listed in Table 7. VIF <5 indicates no significant multicollinearity (VIF ≥5 suggests critical collinearity). Dash (−) denotes variables not included in the specific model.

**Table 7:** Multivariate linear regression analysis of parameter changes.

Dependent variable	Independent variable	β coefficient	95% CI	*P* value
*Δ*FMD (%)	*Δ*BMI (%)	0.32	0.18 to 0.46	0.003
	*Δ*SBP (mmHg)	−0.12	−0.25 to 0.01	0.07
	Age (years)	−0.08	−0.21 to 0.05	0.23
*Δ*SWV (m/s)	*Δ*BMI (%)	−0.41	−0.55 to −0.27	<0.001
	*Δ*FMD (%)	−0.28	−0.39 to −0.17	0.004
	Sex (male)	0.05	−0.08 to 0.18	0.45
*Δ*Ep (kPa)	*Δ*BMI (%)	−0.25	−0.38 to −0.12	0.001
	*Δ*SBP (mmHg)	0.18	0.02 to 0.34	0.04
	Baseline Ep (kPa)	0.10	−0.04 to 0.24	0.16
*Δ*β	*Δ*BMI (%)	−0.19	−0.31 to −0.07	0.008
	*Δ*SBP (mmHg)	0.15	0.01 to 0.29	0.03
	Baseline β	0.06	−0.09 to 0.21	0.44

All models used linear regression with forced entry method. Standardized β coefficients with 95% confidence intervals are reported. Models were adjusted for age, sex, and baseline values of the respective dependent variables as covariates. Model diagnostics confirmed normality of residuals (Shapiro–Wilk *P* > 0.05) and homoscedasticity (Breusch-Pagan *P* > 0.10).

β, standardized regression coefficient; CI, confidence interval; *Δ*, change from baseline.

### 3.6 Adherence-outcome relationships

Dietary adherence averaged 85.4% (SD, 8.2%) for diary completion and 78.2% (SD, 9.5%) for caloric targets. Exercise attendance was 89.6% (SD, 7.3%). Regression models demonstrated that higher dietary adherence predicted greater *Δ*FMD (*β* = 0.24, *P* = 0.01) and *Δ*SWV reduction (*β* = −0.19, *P* = 0.03), while exercise adherence independently associated with *Δ*CD improvement (*β* = 0.18, *P* = 0.04) (Table 8).

**Table 8:** Association between intervention adherence and parameter changes.

Adherence metric	*Δ*FMD	*Δ*SWV	*Δ*CD
Dietary diary completion	*β* = 0.15 (*P* = 0.12)	*β* = −0.11 (*P* = 0.25)	*β* = 0.09 (*P* = 0.35)
Caloric target achievement	*β* = 0.24 (*P* = 0.01)	*β* = −0.19 (*P* = 0.03)	*β* = 0.13 (*P* = 0.18)
Exercise attendance	*β* = 0.11 (*P* = 0.25)	*β* = −0.08 (*P* = 0.41)	*β* = 0.18 (*P* = 0.04)

Linear regression models showing standardized β coefficients (*P*-values) for adherence-outcome relationships after adjustment for age, sex, and baseline BMI. Dietary diary completion: proportion of days with completed food diaries; Caloric achievement: percentage of weeks meeting caloric targets; Exercise attendance: session attendance rate. Significant associations (*P* < 0.05) are bolded.

### 3.7 Sensitivity analysis

After 15 participants who experienced weight rebound (BMI increase ≥ 5%) after intervention were excluded, the improvement in the *Δ*SWV further increased (−0.45 ± 0.07 m/s vs. −0.38 ± 0.09 m/s, *t* = 2.98, *P* = 0.004). The significant increase in the *Δ*FMD (2.9 ± 0.8% vs. 2.5 ± 0.9%, *t* = 2.11, *P* = 0.04) and *Δ*CD (4.9 ± 0.4 kPa vs. 4.5 ± 0.5 kPa, *t* = 2.34, *P* = 0.02) indicated that the main results were robust to weight rebound.

### 3.8 Measurement reliability

The intra-observer and inter-observer reliability for key ultrasound parameters were excellent. For carotid IMT, the intra-observer ICC was 0.92 (95% CI: 0.87–0.95) with a CV of 4.8%, and the inter-observer ICC was 0.88 (95% CI: 0.81–0.93) with a CV of 6.3%. For liver SWV, the intra-observer ICC was 0.94 (95% CI: 0.90–0.97) with a CV of 5.1%, and the inter-observer ICC was 0.90 (95% CI: 0.84–0.94) with a CV of 7.2%.

## 4 Discussion

This study systematically evaluated vascular and hepatic functional changes in children with obesity following multidisciplinary intervention, revealing the positive impact of short-term lifestyle modifications on metabolic health. Although the IMT did not significantly change, improvements in FMD, arterial elasticity parameters, and SWV indicate that functional abnormalities can be reversed early in the intervention, whereas structural remodeling may require longer durations. These findings align with the high vascular plasticity observed in children (13) and suggest prioritizing functional indicators for clinical monitoring to assess intervention efficacy. For example, FMD significantly improved by 6 months postintervention, which is consistent with the findings of Seligman et al., who reported that lifestyle interventions enhance endothelial function in metabolic syndrome patients within 12 weeks (14). Notably, the simultaneous optimization of Ep, β, and CD further supports the sensitivity of arterial elasticity to weight changes, potentially mediated by reduced vascular wall stress and inflammation (15).

The marked reduction in liver stiffness demonstrated that weight control effectively alleviated hepatic steatosis and fibrosis. Studies suggest that obesity itself, independent of metabolic syndrome or insulin resistance, is a primary driver of liver stiffness (16). Sensitivity analysis revealed that the SWV improved after excluding patients with weight rebound, corroborating Reilly et al.'s emphasis on sustained weight loss as pivotal for reversing hepatic pathology (17). Furthermore, the strong correlation between *Δ*BMI and *Δ*SWV (*β* = −0.41) supports the “weight loss threshold hypothesis,” suggesting that a BMI reduction ≥10% may serve as a critical trigger for multiorgan functional improvement (18).

Multivariate regression analysis underscores the central role of weight control. *Δ*BMI independently predicted variations in *Δ*FMD and *Δ*SWV, highlighting its dominance in reversing metabolic abnormalities. However, the independent influence of *Δ*SBP on arterial elasticity parameters (Ep, β) implies that blood pressure management should be combined with weight loss to optimize cardiovascular outcomes in children with baseline hypertension. This aligns with Lurbe et al.'s findings that blood pressure control synergizes with weight reduction to enhance vascular elasticity (19). Additionally, the negative correlation between *Δ*FMD and *Δ*SWV (*r* = −0.46) suggests the existence of a vascular‒liver axis, potentially mediated by the systemic mitigation of inflammation or oxidative stress (20). For example, improvements in endothelial function may reduce the levels of circulating inflammatory factors, indirectly alleviating hepatic lipid accumulation (21). Importantly, the *Δ*FMD-*Δ*SWV and *Δ*Ep/*Δ*β-*Δ*SWV correlations persisted after *Δ*BMI adjustment, suggesting that the vascular-liver axis involves mechanisms beyond weight loss alone, potentially mediated by shared pathways such as systemic inflammation resolution (20) or oxidative stress reduction (21). The attenuation of *Δ*CD/*Δ*AC associations after adjustment implies these parameters may be more directly dependent on adiposity reduction.

Although *Δ*BMI was identified as an independent predictor, it should be interpreted as a surrogate marker reflecting multifaceted physiological improvements rather than a direct causal mechanism. BMI reduction likely reflects synergistic benefits from reduced adiposity, improved metabolic inflammation, and hemodynamic alterations, factors inherently addressed by the multidisciplinary intervention (20, 21). Future mechanistic studies measuring specific mediators (e.g., adipokines, inflammatory cytokines) are warranted to dissect these complex pathways.

The negative correlation between *Δ*FMD (flow-mediated dilation) and *Δ*SWV (shear wave velocity) suggests a link between vascular endothelial function and liver stiffness. Shared inflammatory and oxidative stress pathways, such as reductions in TNF-α and IL-6 following BMI reduction, may improve both FMD and SWV by enhancing endothelial nitric oxide synthase and suppressing hepatic stellate cell activation (22). Biomarkers like hs-CRP and adiponectin could quantify this link, as hs-CRP is associated with both arterial stiffness and liver fibrosis (23). Hemodynamic changes through the hepatic arterial buffer response (HABR) may also connect improvements in systemic vasodilation (FMD) with reduced liver stiffness (24). Finally, gut-derived endotoxins such as LPS may exacerbate both conditions, and interventions restoring gut microbiota may offer beneficial effects (25). Future research should integrate biomarker profiling and advanced imaging to confirm causality.

While retrospective controls (e.g., matched historical cohorts) could theoretically strengthen causal inference, three factors limited this approach: First, temporal variations in diagnostic criteria [e.g., updated pediatric MAFLD guidelines (11)] and ultrasound technologies (ARFI implementation timeline) reduced historical data comparability. Second, insufficient documented pre-intervention parameters (e.g., SWV, CD) in our electronic records precluded propensity scoring. Third, the integrated nature of the multidisciplinary intervention makes it difficult to isolate the effects of its individual components, even with a matched study design. Future studies could employ waitlist-controlled randomization or stepped-wedge designs to reconcile ethical and methodological requirements.

Regarding the nonsignificant IMT change, we acknowledge that structural remodeling may require longer interventions (26). Crucially, the absence of a control group necessitates caution in interpreting temporal changes. However, three factors mitigate confounding: (1) Vascular parameters (FMD, Ep) at baseline were markedly abnormal [vs. pediatric norms (6, 9)], reducing regression-to-mean likelihood; (2) Natural maturation typically improves vascular function minimally [e.g., FMD increases ∼0.5%/year in healthy children (12)], whereas our cohort showed rapid gains (*Δ*FMD = 2.4% in 12 months); (3) Measurement variability was controlled via standardized protocols and high ICCs (0.88–0.94).

Despite promising results, limitations exist. First, the single-center design and modest sample size may limit generalizability. Second, unmeasured confounders such as dietary composition and the gut microbiota could affect regression model interpretability. Third, the lack of long-term follow-up (>2 years) precludes the assessment of IMT changes over time, as structural improvements in adults often require prolonged interventions (26). Fourth, the absence of a control group hinders the isolation of intervention effects from natural growth or environmental factors. Future studies should adopt multicenter designs, incorporate long-term follow-up, and explore molecular mechanisms (e.g., inflammatory biomarker profiling) to elucidate specific pathways underlying the vascular‒liver axis. Fifth, while the multidisciplinary intervention (diet, exercise, and behavioral therapy) was designed as an integrated clinical protocol to reflect real-world practice, we did not statistically isolate component-specific effects due to potential synergistic interactions and limited power for subgroup analyses. Future mechanistic studies with factorial designs are warranted to delineate individual contributions. Finally, although *Δ*BMI statistically predicted improvements in vascular and hepatic parameters, our models did not fully capture unmeasured confounders (e.g., dietary micronutrients, gut microbiota shifts) that may contribute to observed outcomes. This underscores the need to interpret BMI reduction as an integrated marker of metabolic health rather than an isolated driver.

In conclusion, multiparametric ultrasound evaluation provides a reliable tool for dynamic monitoring of intervention efficacy in children with obesity, with weight control remaining the cornerstone of clinical practice. Future research should investigate long-term patterns of arterial structural remodeling and develop individualized strategies for children with distinct baseline profiles, such as intensified blood pressure management for hypertensive patients or behavioral intervention support for families with low adherence.

## Data Availability

The original contributions presented in the study are included in the article/Supplementary Material, further inquiries can be directed to the corresponding author.

## References

[1] Lobstein T Jackson-Leach R Moodie ML Hall KD Gortmaker SL Swinburn BA Child and adolescent obesity: part of a bigger picture. Lancet. (2015) 385(9986):2510–20. 10.1016/S0140-6736(14)61746-3 25703114 PMC4594797

[2] Peking University School of Public Health, Capital Institute of Pediatrics. China Child and Adolescent Obesity Report. Beijing: People’s Medical Publishing House (2017).

[3] Dag N Sinanoglu MS. Evaluation of meniscal elasticity using shear wave elastography in children with obesity and adolescents: a preliminary cross-sectional study [published correction appears in Pediatr Radiol. 2024 Feb;54(2):375. doi: 10.1007/s00247-024-05853-4.]. Pediatr Radiol. (2024) 54(2):293–8. 10.1007/s00247-023-05836-x 38153540

[4] Jebeile H Kelly AS O'Malley G Baur LA. Obesity in children and adolescents: epidemiology, causes, assessment, and management. Lancet Diabetes Endocrinol. (2022) 10(5):351–65. 10.1016/S2213-8587(22)00047-X 35248172 PMC9831747

[5] Schwimmer JB Deutsch R Kahen T Lavine JE Stanley C Behling C. Prevalence of fatty liver in children and adolescents. Pediatrics. (2006) 118(4):1388–93. 10.1542/peds.2006-1212 17015527

[6] Heiss C Rodriguez-Mateos A Bapir M Skene SS Sies H Kelm M. Flow-mediated dilation reference values for evaluation of endothelial function and cardiovascular health. Cardiovasc Res. (2023) 119(1):283–93. 10.1093/cvr/cvac095 35709326

[7] Ravaioli F Dajti E Mantovani A Newsome PN Targher G Colecchia A. Diagnostic accuracy of FibroScan-AST (FAST) score for the noninvasive identification of patients with fibrotic nonalcoholic steatohepatitis: a systematic review and meta-analysis. Gut. (2023) 72(7):1399–409. 10.1136/gutjnl-2022-328689 36599683

[8] Cui X-W Li K-N Yi A-J Wang B Wei Q Wu G-G Ultrasound elastography. Endosc Ultrasound. (2022) 11(4):252–74. 10.4103/EUS-D-21-00151 35532576 PMC9526103

[9] Laurent S Cockcroft J Van Bortel L Boutouyrie P Giannattasio C Hayoz D Expert consensus document on arterial stiffness: methodological issues and clinical applications. Eur Heart J. (2006) 27(21):2588–605. 10.1093/eurheartj/ehl254 17000623

[10] Zhang L Chen J Zhang J Wu W Huang K Chen R Regional disparities in obesity among a heterogeneous population of Chinese children and adolescents [published correction appears in JAMA Netw Open. 2021 Nov 1;4(11):e2138362. doi: 10.1001/jamanetworkopen.2021.38362.]. JAMA Netw Open. (2021) 4(10):e2131040. 10.1001/jamanetworkopen.2021.31040 34698846 PMC8548942

[11] Zhu J-Z Hollis-Hansen K. Clinical guidelines of nonalcoholic fatty liver disease: a systematic review. World J Gastroenterol. (2016) 22(36):8226–33. 10.3748/wjg.v22.i36.8226 27688665 PMC5037092

[12] Akçiçek M Dağ N. Evaluation of hepatic steatosis in children with obesity and adolescents using immune-inflammatory markers and shear wave elastography. J Ultrason. (2025) 25(100):1–6. 10.15557/JoU.2025.0001 39882078 PMC11774258

[13] Urbina EM Khoury PR McCoy C Daniels SR Kimball TR Dolan LM. Cardiac and vascular consequences of prehypertension in youth. J Clin Hypertens. (2011) 13(5):332–42. 10.1111/j.1751-7176.2011.00471.x PMC3092159 21545394

[14] Seligman BGS Polanczyk CA Santos ASB Foppa M Junges M Bonzanini L Intensive practical lifestyle intervention improves endothelial function in metabolic syndrome independent of weight loss: a randomized controlled trial. Metab Clin Exp. (2011) 60(12):1736–40. 10.1016/j.metabol.2011.05.006 21700302

[15] Higashi Y Noma K Yoshizumi M Kihara Y. Endothelial function and oxidative stress in cardiovascular diseases. Circ J. (2009) 73(3):411–8. 10.1253/circj.CJ-08-1102 19194043

[16] Karaman ZF Hatipoğlu N Kardaş F Saraçoğlu S Direk G Kendirci M Identifying the effects of excess weight, metabolic syndrome and insulin resistance on liver stiffness using ultrasound elastography in children. Turk J Pediatr. (2022) 64(4):671–82. 10.24953/turkjped.2021.1876 36082641

[17] Reilly JJ Kelly J. Long-term impact of overweight and obesity in childhood and adolescence on morbidity and premature mortality in adulthood: systematic review. Int J Obes. (2011) 35(7):891–8. 10.1038/ijo.2010.222 20975725

[18] Kelly AS Arslanian S Hesse D Iversen AT Körner A Schmidt S Reducing BMI below the obesity threshold in adolescents treated with once-weekly subcutaneous semaglutide 2.4 mg. Obesity (Silver Spring). (2023) 31(8):2139–49. 10.1002/oby.23808 37196421

[19] Lurbe E Agabiti-Rosei E Cruickshank JK Dominiczak A Erdine S Hirth A 2016 European society of hypertension guidelines for the management of high blood pressure in children and adolescents. J Hypertens. (2016) 34(10):1887–920. 10.1097/HJH.0000000000001039 27467768

[20] Targher G Byrne CD Lonardo A Zoppini G Barbui C. Nonalcoholic fatty liver disease and risk of incident cardiovascular disease: a meta-analysis. J Hepatol. (2016) 65(3):589–600. 10.1016/j.jhep.2016.05.013 27212244

[21] Virdis A Duranti E Rossi C Dell'Agnello U Santini E Anselmino M Tumor necrosis factor-alpha participates on the endothelin-1/nitric oxide imbalance in small arteries from obese patients: role of perivascular adipose tissue. Eur Heart J. (2015) 36(13):784–94. 10.1093/eurheartj/ehu072 24578389

[22] Francque SM van der Graaff D Kwanten WJ. Non-alcoholic fatty liver disease and cardiovascular risk: pathophysiological mechanisms and implications. J Hepatol. (2016) 65(2):425–35. 10.1016/j.jhep.2016.04.005 27091791

[23] Petta S Maida M Macaluso FS Di Marco V Cammà C Cabibi D The severity of steatosis influences liver stiffness measurement in patients with nonalcoholic fatty liver disease. Hepatology. (2015) 62(4):1101–10. 10.1002/hep.27844 25991038

[24] Feng H Liu T Wang M Wang Z. Experimental study of hepatic arterial buffer response after occluding hepatic portal vein and superior mesentery artery. J Bethune Mil Med Coll. (2003) 1(4):195–7.

[25] Tripathi A Debelius J Brenner DA Karin M Loomba R Schnabl B The gut-liver axis and the intersection with the microbiome [published correction appears in Nat Rev Gastroenterol Hepatol. 2018 Dec;15(12):785. doi: 10.1038/s41575-018-0031-8.]. Nat Rev Gastroenterol Hepatol. (2018) 15(7):397–411. 10.1038/s41575-018-0011-z 29785003 PMC7133393

[26] O’Leary DH Polak JF. Intima-media thickness: a tool for atherosclerosis imaging and event prediction. Am J Cardiol. (2002) 90(10C):18 L–21 L. 10.1016/S0002-9149(02)02957-0 12459422

