# Supplementary material for: Longitudinal study on ultrasound assessment of peripheral arterial elasticity and liver stiffness for evaluating clinical intervention efficacy in children with obesity
Source: Front Pediatr. 2025 Nov 21;13:1604576. doi: 10.3389/fped.2025.1604576 (PMC12678271; doi:10.3389/fped.2025.1604576)
Supplement: Supplementary file 1 [file Table1.docx]

### ****Supplementary Table 1.** Multidisciplinary intervention protocol**

| **Category** | **Description** | **Implementer** | **Frequency/Duration** | **Tools/Methods** | **Adaptations** |
| --- | --- | --- | --- | --- | --- |
| **Dietary Control** | Individualized low-calorie diet plan: 20% reduction in daily caloric intake, optimized macronutrient composition (high fiber, lean protein, unsaturated fats; limited added sugars and saturated fats). | Registered dietitian | Daily for 12 months | Dietary diary; nutrition analysis software (e.g., "Dietary Treasure") | Dynamic adjustments based on monthly weight changes and metabolic assessments. |
| **Exercise Program** | Moderate-intensity aerobic exercise (e.g., brisk walking, swimming, cycling), supervised to maintain target heart rate ≥140 bpm. Exercise intensity progressively increased based on baseline fitness levels. | Rehabilitation team | 5 sessions/week, 60 min | Heart rate monitors (e.g., Fitbit Charge 5); exercise logs | Phase-wise intensity progression (e.g., Week 1–4: 50% max HR; Week 5–12: 70%). |
| **Behavioral Therapy** | Family-involved cognitive-behavioral therapy (CBT) sessions focused on self-monitoring, goal-setting, stress management, and environmental modification. Interactive methods (role-playing, simulations) applied. | Behavioral therapist | Monthly sessions | Group workshops; homework assignments (e.g., "Healthy Challenge Week Planner") | Tailored feedback via WeChat follow-ups to address adherence barriers. |
| **Support & Monitoring** | Monthly teleconsultations and real-time guidance via instant messaging (WeChat) to reinforce compliance and troubleshoot challenges. | Research coordinator | Monthly | Electronic health records (EMR); encrypted communication platforms | Customized strategies for high-risk subgroups (e.g., motivational interviewing). |

**Notes**: **Abbreviations**: HR = Heart rate; CBT = Cognitive-behavioral therapy; EMR = Electronic medical records.
