# Supplementary material for: Longitudinal study on ultrasound assessment of peripheral arterial elasticity and liver stiffness for evaluating clinical intervention efficacy in children with obesity
Source: Front Pediatr. 2025 Nov 21;13:1604576. doi: 10.3389/fped.2025.1604576 (PMC12678271; doi:10.3389/fped.2025.1604576)
Supplement: Supplementary file 2 [file Datasheet1.docx]

B

A


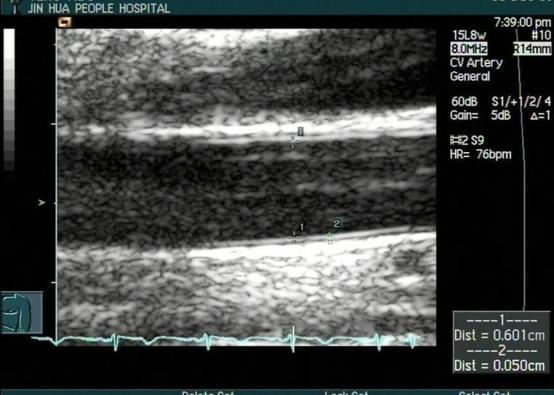

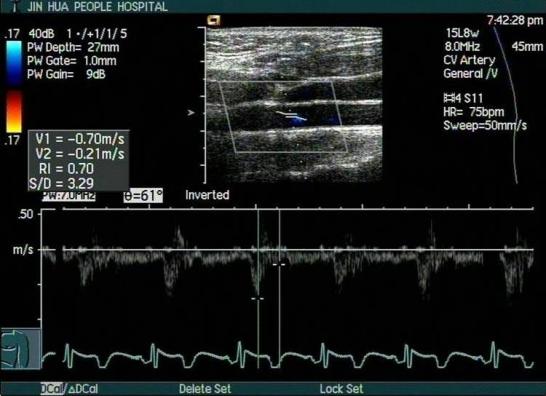


Supplementary Figure 1. Ultrasound assessment of carotid artery parameters in obese children. (A) B-mode ultrasound measurement of carotid artery intima-media thickness (IMT = 0.050 cm) and lumen diameter (0.601 cm). (B) Doppler spectral analysis of carotid artery blood flow showing velocity parameters (V1 = -0.70 m/s, V2 = -0.21 m/s) and resistance index (RI = 0.70). These measurements were used to calculate vascular elasticity parameters including elasticity modulus (Ep), stiffness index (β), distensibility coefficient (DC), and arterial compliance (AC) for longitudinal monitoring.

A

B


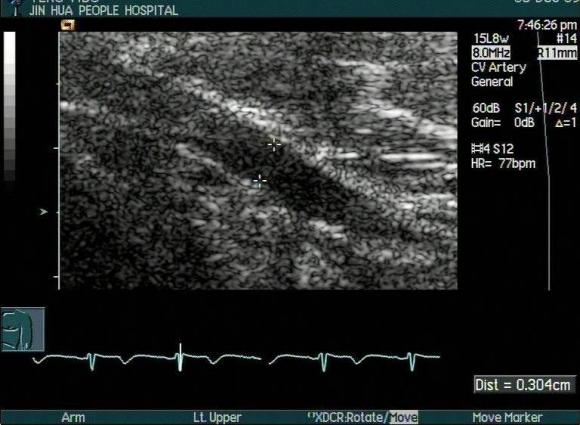

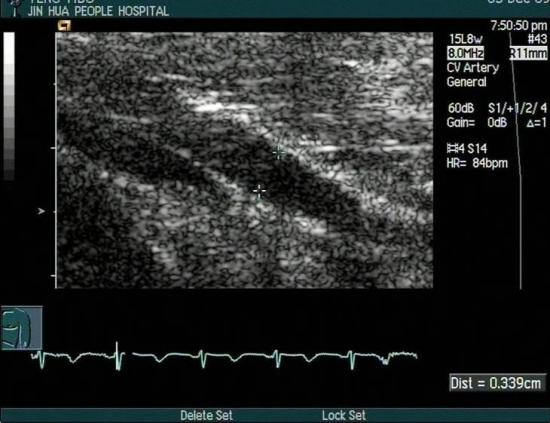


Supplementary Figure 2. Carotid artery ultrasound measurements for vascular elasticity assessment. (A) Measurement of carotid artery diameter (0.304 cm) during diastole. (B) Measurement of carotid artery diameter (0.339 cm) during systole. These measurements, along with blood flow parameters, were used to calculate vascular elasticity indices including elasticity modulus (Ep), stiffness index (β), distensibility coefficient (DC), and arterial compliance (AC) for longitudinal comparison during the intervention period.
